# N-Halamine Biocidal Materials with Superior Antimicrobial Efficacies for Wound Dressings

**DOI:** 10.3390/molecules22101582

**Published:** 2017-09-21

**Authors:** Buket Demir, Roy M. Broughton, Mingyu Qiao, Tung-Shi Huang, S. D. Worley

**Affiliations:** 1 Department of Chemistry and Biochemistry, Auburn University, Auburn, AL 36849, USA; bzd0003@tigermail.auburn.edu; 2 Center for Polymers and Advanced Composites, Department of Mechanical Engineering, Auburn University, Auburn, AL 36849, USA; brougrm@auburn.edu; 3 Department of Poultry Science, Auburn University, Auburn, AL 36849, USA; mzq0001@tigermail.auburn.edu (M.Q.); huangtu@auburn.edu (T.-S.H.)

**Keywords:** N-halamine, antimicrobial efficacies, medical wound dressings, antibacterial materials

## Abstract

This work demonstrated the successful application of N-halamine technology for wound dressings rendered antimicrobial by facile and inexpensive processes. Four N-halamine compounds, which possess different functional groups and chemistry, were synthesized. The N-halamine compounds, which contained oxidative chlorine, the source of antimicrobial activity, were impregnated into or coated onto standard non-antimicrobial wound dressings. N-halamine-employed wound dressings inactivated about 6 to 7 logs of *Staphylococcus aureus* and *Pseudomonas aeruginosa* bacteria in brief periods of contact time. Moreover, the N-halamine-modified wound dressings showed superior antimicrobial efficacies when compared to commercially available silver wound dressings. Zone of inhibition tests revealed that there was no significant leaching of the oxidative chlorine from the materials, and inactivation of bacteria occurred by direct contact. Shelf life stability tests showed that the dressings were stable to loss of oxidative chlorine when they were stored for 6 months in dark environmental conditions. They also remained stable under florescent lighting for up to 2 months of storage. They could be stored in opaque packaging to improve their shelf life stabilities. In vitro skin irritation testing was performed using a three-dimensional human reconstructed tissue model (EpiDerm™). No potential skin irritation was observed. In vitro cytocompatibility was also evaluated. These results indicate that N-halamine wound dressings potentially can be employed to prevent infections, while at the same time improving the healing process by eliminating undesired bacterial growth.

## 1. Introduction

Wound care is of great importance, and it can be a burden for chronic wound treatments caused by diseases such as ulcers, vascular and diabetes, and acute wounds such as burns and post surgical wounds where the healing process is extended, or for wounds which fail to heal. As a result, infection control becomes a major problem [1]. These types of wounds can easily become infected and life threating if not treated properly [2,3]. In addition to loss of function, decreased quality of life and morbidity, costs associated with chronic wound care represent a significant burden to healthcare systems worldwide and are expected to continue to rise. Using antibiotics along with dressings is a common practice in wound treatments. However, there is an increasing concern in an incidence of rise of antibiotic resistant microorganisms caused by indiscriminate usage of antibiotics, presenting serious health concerns worldwide [4,5,6]. As a result, antiseptic treatments are becoming increasingly necessary in controlling bacterial contamination in health care systems [7]. Antimicrobial materials (biocides) have been used as antiseptic treatments for centuries and are regaining their importance due to their advantages over antibiotics. In addition, unlike antibiotics, biocides are straight-forward in use, available without prescription and less expensive than antibiotics [8].

Numerous antimicrobial agents have been developed and explored in an effort to inhibit the growth of bacteria and to prevent infections in health care applications [9]. Among these agents are metal ions, silver nanoparticles, biguanides, and quaternary ammonium compounds, which are commonly used biocides for wound dressing applications [10,11]. However, technologies utilizing biocidal compounds, such as silver nanoparticles, triclosan and biguanides, that are known to cause environmental and toxicity problems, have been employed recently [12]. High levels of exposure to these agents raise health concerns. Research has shown that silver nanoparticles were released from textiles during washing cycles and bio-accumulated into the environment, which elevated the exposure of humans to silver metal [13,14]. In addition to these toxicity issues, it has been well documented that some biocides such as silver have shown bacterial resistance [15,16]. Moreover, the antimicrobial efficacy of silver dressings is generally poor. For example, silver-incorporated materials can require about 24 h of contact time in order to deactivate pathogenic microorganisms [17]. It has also been noted that rapid inactivation of bacteria is necessary for an ideal wound dressing in order to prevent infections and control cross-contamination [2].

Since the discovery of the halogens chlorine and iodine, they have been used for centuries as disinfectants. The main limitation of using the halogens as disinfectants is their stability. This problem was solved with development of organic N-halamines. N-halamines consist of a diverse class of biocides, which are characterized in the form of amines, amides or imides. These functional groups can form covalent bonds with halogens where the halogen ion (chlorine, bromine or iodine) is stabilized as oxidative halogen, which is biocidal.

Among other biocides used for antimicrobial applications, N-halamines offer many benefits. For over three decades extensive work on N-halamine antimicrobial compounds has been progressing in the Worley laboratories and elsewhere [18,19,20,21,22,23,24,25,26,27,28,29,30,31,32,33,34,35,36,37,38,39,40,41,42,43,44,45,46]. They are generally the most effective antimicrobial materials due to their rapid inactivation efficacies against a broad spectrum of microorganisms (Gram-positive and Gram-negative bacteria, yeasts, fungi and viruses) [20,31,32,33,34,35]. Depending on the chemical structure, they have long-term halogen stability and antimicrobial functionality. Their most unique characteristic is that once oxidative halogen inactivates the microorganisms and is exhausted, they can be recharged and therefore can continue the inactivation of pathogens after being recharged [33,36,37,38]. They are relatively inexpensive, and they are generally non-leaching when they are covalently attached onto surfaces [39]. The mechanism of action of stable N-halamines involves a direct contact of the N-halamine with the bacterial cell during which the oxidative halogen is transferred to the cell. The molecule does not dissociate into free oxidative chlorine before transfer to the cell [40]. In addition, it has been reported that the N-halamines do not show any evidence of bacterial resistance [41].

N-halamines can be employed in a variety of applications. N-halamine molecules can be incorporated into various substrates [21,34], modified to produce desirable functional groups that allow covalent attachment to surfaces by chemical bonds or ionic interactions [27,36,42,43,44], impregnated into fibrous materials [28], polymerized onto surfaces or used as monomers or polymers as disinfectants [18,20,43].

Today most wound dressings lack long term antimicrobial function, stability, and rapid disinfection ability, and may have a risk of resistance to bacteria. There is a need for development of new, inexpensive, biodegradable, non-leaching, less toxic, environmentally friendly antimicrobial wound dressings. This could potentially be achieved when N-halamine compounds are incorporated as biocides into the wound dressings. In an effort to address and overcome the challenges and limitations, the current research has primarily focused on developing an ideal antimicrobial wound dressing with use of N-halamine biocides. In this regard four different N-halamine compounds have been synthesized and incorporated into wound dressing materials. Antimicrobial efficacies, stabilities, skin sensitivities and cytotoxicities of the N-halamine-coated wound dressing materials have been evaluated.

## 2. Results and Discussions

### 2.1. Characterization of the Synthesized Compounds

**Scheme 1:**
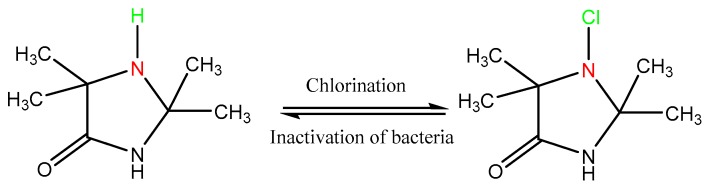
1-chloro-2,2,5,5-tetramethyl-4-imidazolidinone (MC) synthesis route by chlorination of 2,2,5,5-tetramethyl-1,3-imidazolidinone (TMIO) precursor.

**Scheme 2:**
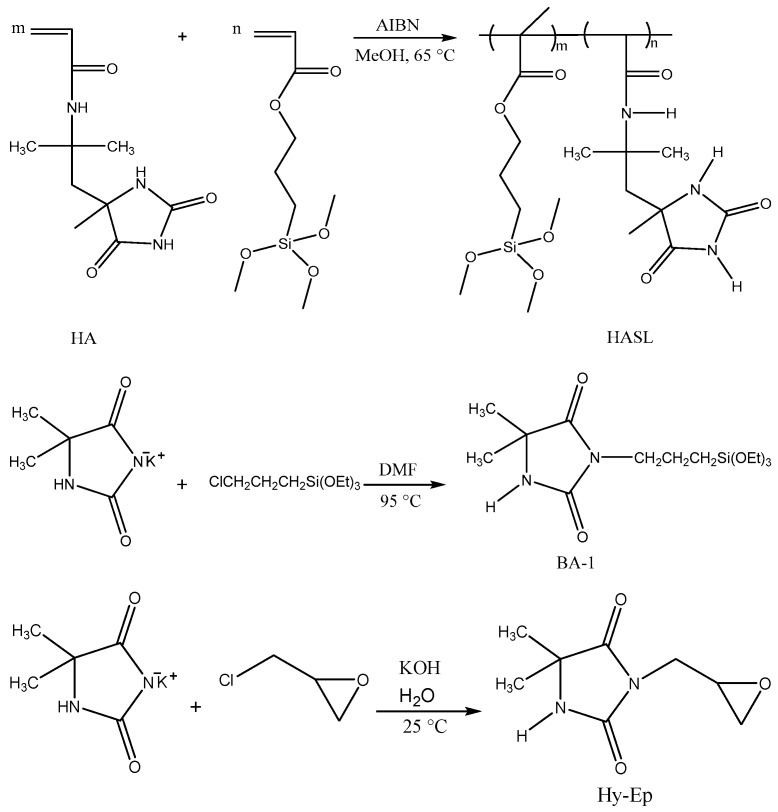
Schematic synthesis routes of 2-acrylamido-2-methyl-1-(5-methylhydantoinyl)propane/siloxane functional methacrylate (HASL) copolymer, BA-1 and Hy-Ep monomer precursors.

The methods applied in this work were straightforward and have been discussed in the Materials and Methods section in detail. The 1-chloro-2,2,5,5-tetramethyl-4-imidazolidinone (MC) compound was synthesized by halogenation of its precursor 2,2,5,5-tetramethyl-1,3-imidazolidinone (TMIO) (Scheme 1); the precursor was prepared as described previously [20]. Synthesis routes of the precursor products are illustrated in Scheme 2. The 3-glycidyl-5,5-dimethylhydantoin and 3-triethoxysilylpropyl-5,5-dimethylhydantoin were synthesized by the nucleophilic reaction of 3-chloropropyltriethoxysilane and epichlorohydrin, respectively, with the potassium salt of 5,5-dimethylhydantoin. HASL copolymer was obtained by free radical polymerization of 2-acrylamido-2-methyl-1-(5-methylhydantoinyl)propane (HA) and a siloxane functional methacrylate (SL). These three precursors were rendered antimicrobial by exposure to dilute household bleach after they were attached to wound dressings.

Information concerning the application of the N-halamine compound or its precursor onto the wound dressings is crucial since the type of N–Cl bond is important for the chlorine stability, and especially for the toxicity and skin sensitivity data. The structures of the N-halamine precursors were confirmed by NMR and FTIR characterization. NMR data for the synthesized compounds have been included in the Materials and Methods Section and Supplementary Materials.

### 2.2. Preparation and Characterization of the Coatings

**Figure 1:**
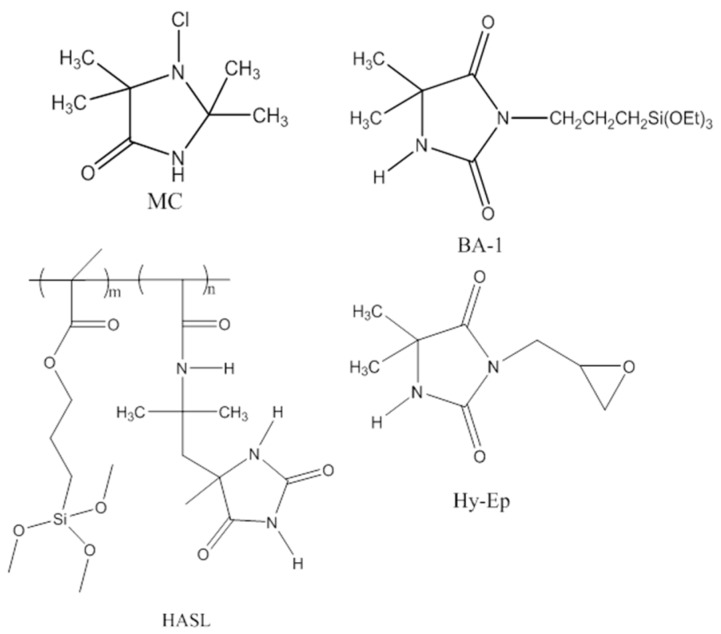
Synthesized N-halamine compound or precursors.

Wound dressings were prepared by impregnating N-halamine or its precursor moieties into or onto the wound dressings by coating processes. Structures of the synthesized N-halamine or precursor compounds used for the wound dressing application are shown in Figure 1. In order to obtain optimum chlorine loadings, wound dressings were treated in N-halamine or precursor coating solutions prepared at different concentrations depending upon the N-halamine compound desired. Due to the difference in the chemical structure and available N–H sites in each compound, the number of bound chlorine atoms could differ. Therefore, the coating concentrations of HASL, BA-1, Hy-Ep and MC were adjusted to obtain approximately the same number of chlorine atoms on each of the N-halamine-treated wound dressings. After the coating and curing processes, three of the synthesized N-halamine precursors of BA-1, HASL and Hy-Ep were covalently bound onto the wound dressings through siloxane or epoxy moieties as represented in the precursor structures, respectively. BA-1, HASL and Hy-Ep functionalized wound dressings were then rendered antimicrobial by reaction of the N–H sites with free chlorine during a dilute bleach chlorination process. The MC compound included the N-chloramine moiety prior to the impregnation process. It was not covalently bound to the fibrous matrix of the wound dressing; rather, it was bound by electrostatic attraction, but it could not be mechanically removed from the dressing. After the chlorination process, the presence of oxidative chlorine bonded to the N-halamine precursors was confirmed by analytical titration of the N-halamine treated samples. The weight percent of oxidative chlorine amount on the samples of the MC, BA-1-Cl, Hy-Ep-Cl and HASL-Cl coated wound dressings to be used in the antimicrobial testing procedure were measured to be 0.22 wt % Cl^+^, 0.21 wt % Cl^+^, 0.19 wt % Cl^+^ and 0.23 wt % Cl^+^, respectively.

**Figure 2:**
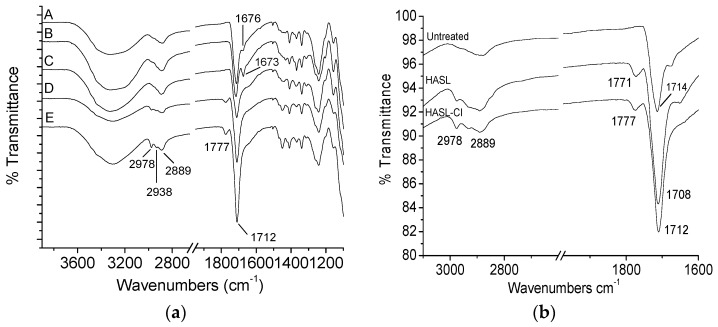
Fourier Transform Infrared (FTIR) specta of untreated and N-halamine treated wound dressings. (**a**) FTIR spectra of A: untreated, B: MC-treated, C: Hy-Ep-Cl-treated, D: BA-1-Cl-treated and E: HASL-Cl-treated dressings (spectra taken after chlorination of Hy-Ep, BA-1 and HASL-treated wound dressings); (**b**) FTIR spectra of untreated, HASL-treated and HASL-Cl-treated dressings.

Surface characterization of untreated controls, MC, BA-1, HASL and Hy-Ep-treated and chlorinated-treated (BA-1-Cl, HASL-Cl, Hy-Ep-Cl) was performed by Fourier Transform Infrared (FTIR) spectroscopy. FTIR spectra of N-halamine-treated and halogen-activated wound dressings are shown in Figure 2a. In this spectra data collected from N-halamine wound dressings after the chlorination process can be compared to the data collected from the untreated native wound dressing (A). MC, Hy-Ep-Cl, BA-1-Cl and HASL-Cl-treated wound dressing spectra are demonstrated as B, C, D, E, respectively. The characteristic carbonyl stretching bands of the hydantoin ring (imide and amide groups) of the compounds were observed at about 1777 and 1712 cm^−1^. The band at 1673 cm^−1^ of the halogen-activated N-halamine dressing (B) was suggestive that the MC compound was successfully impregnated into the dressing (an additional spectrum of the MC compound is included in Supplementary Materials Figure S5). Similar to the other N-halamine treated dressings, the bands due to the hydantoin ring were also present in the FTIR spectra of HASL and HASL-Cl (Figure 2b). After chlorination, a slight shift to a higher wavenumber of the vibrational stretching bands of the hydantoin carbonyl groups of HASL-Cl was observed due to transformation of N–H bonds present in HASL to N–Cl bonds. This data showed that the HASL polymer was bound to the fibrous matrix and remained bound after the chlorination process. In addition, no new bands appeared after the halogenation process when compared to the spectrum of the untreated dressing, indicating that the halogenation process did not affect the structure of the native dressing. Similar results to those seen in Figure 2b for the HASL dressings were observed for the other Hy-Ep and BA-1 coated dressings for which the transformation of the N–H bond to an N–Cl bond was also detected.

### 2.3. Shelf Life Stability 

Shelf life (storage) stability of the BA-1-Cl, HASL-Cl, Hy-Ep-Cl and MC-treated wound dressings are shown in Table 1. Bound chlorine in MC-treated dressings remained for 24 weeks of storage in a dark environment. BA-1-Cl-treated dressings were stored in dark environmental conditions and retained most of their initial chlorine loadings for 10 weeks. However, HASL-Cl and Hy-Ep-Cl-treated dressings were somewhat less stable than MC and BA-1-Cl-treated dressings. BA-1-Cl-treated dressings lost only 24% of chlorine after 6 months of storage in darkness. It was observed that Hy-Ep-Cl and HASL-Cl-treated dressings retained 65% and 58% of their chlorine amounts after 16 weeks of storage in a dark environment. The variation in the chlorine loading data for each of the single N-halamine treatments shown in Table 1 could be attributed to different fiber and structural uniformity of the commercial wound dressing samples used. Therefore the bonded chlorine amount could slightly differ from sample to sample. However, the chlorine bond stability changed between N-halamine treated samples, MC being the most stable of the four N-halamines. There was no significant change or loss in bound chlorine when MC-treated samples were stored in darkness. These results revealed that the MC-treated wound dressing samples had excellent storage stability and did not show any significant loss of oxidative chlorine loading during storage.

Halogen stability differs according to N–Cl chemistry. The MC compound showed the highest stability because of its N-chloramine chemistry (the Cl is bonded to an amine N); whereas, Hy-Ep-Cl, BA-1-Cl and HASL-Cl include N-chloramide and N-chlorimide moieties for which the N–Cl dissociation constants are higher. As a result, dressings coated with these N-halamines showed some less stability over time.

It should also be noted that the N–Cl bond dissociation increased with exposure to UVA photons. When stored under florescent light, MC samples lost only 32% of the oxidative chlorine over 24 weeks of storage. Compared to previous UVA light stability data for N-halamine compounds, it can be concluded that the MC compound is the most stable N-halamine compound yet developed in these laboratories [20].

### 2.4. Antimicrobial Efficacy Testing

In both types of the antimicrobial tests (antimicrobial efficacy and zone of inhibition) two species of bacteria, Gram-positive *Staphylococcus aureus* ATCC 6538 and Gram-negative *Pseudomonas aeruginosa* ATCC 27853, were employed. Precursor-treated (Hy-Ep, BA-1, HASL) and untreated native dressings were used as controls. Those control samples, N-halamine-treated ones (Hy-Ep-Cl, BA-1-Cl, HASL-Cl and MC) and commercial antimicrobial (silver alginate and polybiagunide (PHMB)) dressings were evaluated for antimicrobial efficacy. Each test was repeated at least twice on different days, and the results are shown below in Table 2, Table 3, Table 4, Table 5 and Table 6.

As can be seen, all of the compounds showed efficacy against the two bacteria (*S. aureus* and *P. aeruginosa*) at populations of about 10^6^ to 10^7^ CFU (colony forming units). Untreated native and un-chlorinated-precursor-treated (control) samples exhibited much lower reductions, even after 60 min of contact time (Table 2, Table 3, Table 4 and Table 5). These reductions were due to adherence of live bacteria to the fibers, not to inactivation of bacteria. MC-treated dressings appear to be superior as they inactivated 6 logs of *S. aureus* in about a maximum of 15 min and inactivated 5.7 to 7.4 logs of *P. aeruginosa* in about a maximum of 30 min of contact time (Table 2). In contrast to the MC results, after 60 min of contact time, BA-1-Cl-treated samples exhibited a complete inactivation of about 6 logs of *S. aureus* and *P. aeruginosa* (Table 3). The log reduction for the BA-1-Cl-treated samples varied from experiment to experiment, but it is not unusual to observe such inconsistencies in repeated experiments, especially when a larger inoculum of bacteria (10^7^ CFU) was used. Hy-Ep-Cl-treated dressings exhibited similar results to BA-1-Cl coated samples; the samples provided about 6 log reductions against *S. aureus* and *P. aeruginosa* in 60 min of contact time (Table 4). HASL-Cl-treated samples provided complete kill of 6 logs of *S. aureus* in 15 min. However, in one of the experiments complete inactivation of *S. aureus* was observed after 30 min of contact time. Similar to MC-treated samples, only 30 min of contact time was required to inactivate 6 logs of *P. aeruginosa* (Table 5). The variability in the data is as expected due to the difficulty of performing the experiments. One bacterial CFU trapped in the wound dressing away from any chlorine can lead to these observations. It can be concluded from these results that all of the N-halamine compounds could be effective antimicrobial materials in wound dressings. In contrast, for a commercial silver alginate dressing we obtained less than 1 log of inactivation for both bacterial species in 30 min of contact using the same experimental sandwich test. PHMB-treated dressings showed poor inactivation efficacy against Gram-negative *P. aeruginosa* compared to all four N-halamine-treated dressings. Only in one experiment were they able to inactivate about 7 logs of *S. aureus* in 15 min (Table 6). It was observed that N-halamine dressings showed rapid inactivation and superior antimicrobial efficacy when compared to silver and biguanide dressings.

### 2.5. Zone of Inhibition Test

In this test, as for the sandwich test, two species of bacteria, Gram-positive *Staphylococcus aureus* ATCC 6538 and Gram-negative *Pseudomonas aeruginosa* ATCC 27853, were employed to determine the zone of inhibition. Un-chlorinated precursor-treated (Hy-Ep, BA-1, HASL) and untreated native samples were used as controls, and N-halamine-treated ones (Hy-Ep-Cl, BA-1-Cl, HASL-Cl and MC) were used as test samples against the bacteria. In contrast to the sandwich antimicrobial efficacy test, zone of inhibition is a qualitative test method. A zone of inhibition occurs when a material prevents the growth of bacteria. The diameter of the zone determines the antimicrobial property when the biocidal agent is released from the treated surface. Therefore, a clear zone where bacteria growth was prevented can be only observed when active compound of the biocidal agent leaches out from the test materials. It can be concluded from zone of inhibition testing (Kirby-Bauer testing technique) for the three compounds (MC, BA-1-Cl and Hy-Ep-Cl) that there was no significant leaching of the compounds or oxidative chlorine from the wound dressing samples, as no halo was observed around the tested disks (Supplementary Materials, Figure S6). However, a slight zone was observed around the edges of the HASL-Cl-coated disks. This could be attributed to less stability of the aliphatic amide N–Cl bonds represented in the HASL-Cl compound. Zone of inhibition results revealed that strong N–Cl bonding occurred and suggested that the killing mechanism of the N-halamine compounds occurred by direct contact of the N–Cl group with bacteria on the wound dressing. Thus leaching of the antimicrobial from the wound dressing should not create a regulatory problem.

### 2.6. Cytotoxicity of N-Halamine-Treated Wound Dressings

**Figure 3:**
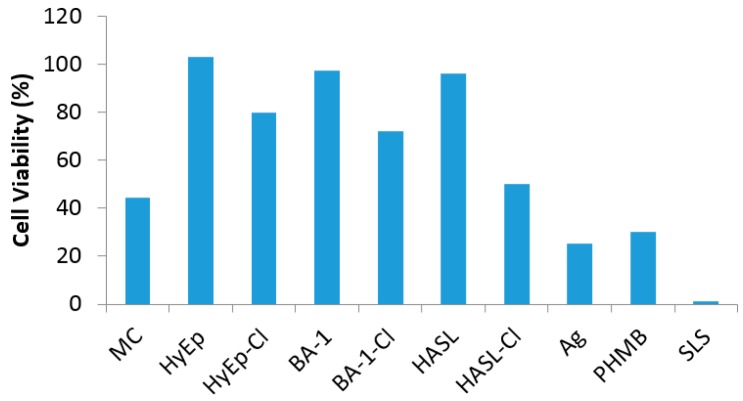
Cell viability analysis (MTT assay) on mouse fibroblast (NIH-3T3) of N-halamine precursor-coated and N-halamine-coated, and commercial silver alginate (Ag) and polybiguanide (PHMB) wound dressing test specimens.

The effect of precursor N-halamine-coated and N-halogen-activated wound dressings on cell survivability on the fibroblast (NIH-3T3) cells was evaluated (Figure 3). Cell viability % was calculated with respect to untreated control test samples. A 5% sodium lauryl sulfate solution (SLS) was used as the positive control, which was presumed to have high toxicity levels. Only 1% or less cell viability was observed indicating the toxicity effects of SLS to cells. Un-chlorinated samples (Hy-Ep, BA-1 and HASL) showed an average of more than 80% of cell viability. However, N-halamine-treated (MC, HyEp-Cl, BA-1-Cl, HASl-Cl) samples showed 60% or less cell viability. Compared to N-halamine dressings, commercial silver (Ag) and polybiagunide (PHMB) wound dressings showed only 24% and 29% cell viability, respectively, which corresponds to their toxicity to cells. These results suggested that N-halamine compounds do not significantly inhibit the cell viability; however, some Cl^+^ could dissociate from the N-halogonated samples into the culture medium over an extended period of time and could interfere with the cell viability. Decreasing the percentage of the Cl^+^ in dressings and test medium should minimize the effects. This could be achieved without affecting the antimicrobial efficacy of the dressings. It has been previously reported that 0.04 wt % Cl^+^ loading would be sufficient for antimicrobial efficacy [28,39].

### 2.7. In Vitro Skin Irritation

**Figure 4:**
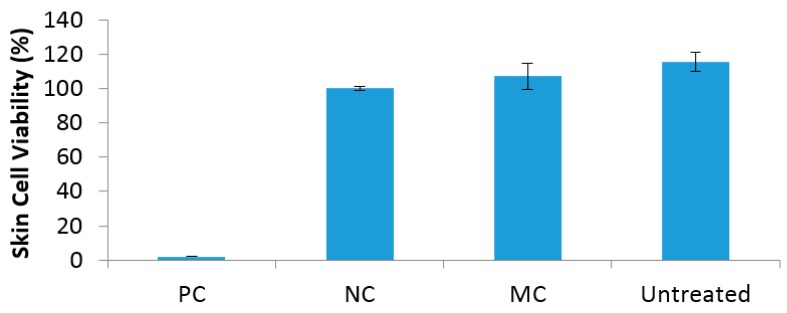
Skin irritation of PC (positive control), NC (negative control), MC-treated wound dressing specimen and untreated wound dressing specimen tested in an in vitro reconstructed human epidermis prediction model EpiDerm™.

In vitro skin irritation of the MC-impregnated wound dressings was evaluated using a reconstructed human epidermal (RHE) model (ISO10993-10). Compared to all of the other N-halamine-treated dressings, MC-treated samples showed the highest efficacy and chlorine stability. Due to the results obtained from previous tests and the limitations in number of samples available for this test, MC-treated dressings were selected as test samples for skin irritation evaluation. For each chemical treated epidermis, % viability was expressed as [(OD_sample_ − OD_blank_)/(OD_control_ − OD_blank_)] × 100. The irritation potential of the MC compound was predicted to be a non-irritant, where 100% of skin epidermis cells survived (Figure 4). In contrast, the positive control (5% sodium lauryl sulfate solution) caused destruction of cells resulting in no epidermis cell viability. As expected, native untreated sample and the negative control (D-PBS buffer solution) showed no irritation potential where 100% cell viability was observed, similar to MC-treated epidermis. It has been reported as a guideline that compounds are considered as non-irritant when the cell viability is more than 50% relative to the negative control. Hence, the results demonstrated that this was the case for N-halamine monomer MC-treated wound dressings.

## 3. Materials and Methods

### 3.1. Materials and Instrumentation

5,5-dimethylhydantoin, 3-chloropropyltriethoxysilane, 2-acrylamido-2-methyl-4-pentanone, epichlorohydrin, potassium cyanide, ammonium carbonate and most of the other chemicals were purchased from Acros Organics (Morris Plains, NJ, USA) and VWR Inc. (Radnor, PA, USA). All chemicals were used without further purification. Band-Aid^®^ brand non-antimicrobial wound dressing materials were obtained from Johnson & Johnson Co. (Skillman, NJ, USA). CVS/pharmacy^TM^ brand antimicrobial sterile silver alginate dressings and antibacterial sterile PHMB gauze pads (dressings) were purchased from CVS Pharmacy Inc. (Woonsocket, RI, USA). Clorox^®^ brand (Clorox, Inc., Oakland, CA, USA) household bleach (8.25% of NaOCl) was used for the chlorination process. Bacterial cultures of *S. aureus* ATCC 6538 and *P. aeruginosa* ATCC 27853 were purchased from American Type Culture Collection (Rockville, MD, USA), and Trypticase soy agar was obtained from Difco Laboratories (Detroit, MI, USA). Newborn calf serum (NCS) was purchased from HyClone Laboratories (South Logan, UT, USA). Dulbecco’s modification of eagle’s medium (DMEM) without L-glutamine (4.5 mg/L glucose) was obtained from Lonza Inc. (Walkerville, MD, USA). 3-(4,5-dimethylthiazol-2-yl)-2,5-diphenyltetrazolium bromide (MTT) was purchased from Alfa Aesar (Ward Hill, MA, USA). Mouse embryonic fibroblast cells BALB/c 3T3 clone A31 (ATCC^®^ CCL163™) were purchased from the American Type Culture Collection (ATCC; Manassas, VA, USA). A reconstructed human epidermal model EpiDerm™ kit (EPI-200-SIT) was purchased from MatTek Corporation (Ashland, MA, USA).

NMR spectra were obtained using a Bruker Avance 400 MHz spectrometer (Bruker, Inc., Billerica, MA, USA); ^1^H-NMR spectra were recorded with 16 scans. FTIR data were obtained with a Model Spectrum 400 (Perkin Elmer Co., Waltham, MA, USA) and recorded with 32 scans at 4 cm^−1^ resolution.

### 3.2. Synthesis of N-Halamine Compounds

#### 3.2.1. Synthesis of 3-Glycidyl-5,5-dimethylhydantoin (Hy-Ep)

3-Glycidyl-5,5-dimethylhydantoin was prepared according to a procedure outlined previously [34,35]. Briefly, 0.05 mol of 5,5-dimethylhydantoin was converted into its sodium salt by stirring in an equimolar quantity of NaOH in aqueous solution at ambient temperature for 10 min. Hy-Ep was synthesized by subsequent addition of epichlorohydrin (0.05 mol) into the mixture and stirring at ambient temperature for 10 h. After reaction, water was removed by vacuum evaporation, and the hydantoin epoxide derivative was dissolved in acetone. Byproduct sodium chloride was removed by filtration. The final product was obtained after removing acetone. The yield obtained was 50% after purification. For analytical testing the hydantoin epoxide derivative could further be purified by column chromatography for spectroscopic analysis. ^1^H-NMR (d-CDCl_3_): δ 1.43 (s, 6H), 2.87–2.97 (m, 2H), 3.24 (m, 1H), 3.36–3.59 (m, 2H), 4.16 (s, 1H) (Figure S1 in Supplementary Materials). However, the purification process was not necessary for coating onto the fibrous materials (wound dressings) used in this study. After coating and covalent attachment onto the surface, unreacted precursor materials were washed off by an extensive cleaning and washing procedure; therefore, the crude product was used for coating onto the wound dressing material. 

#### 3.2.2. Synthesis of 3-Triethoxysilylpropyl-5,5-dimethylhydantoin (BA-1)

3-Triethoxysilylpropyl-5,5-dimethylhydantoin (BA-1) was prepared according to the procedure outlined in [39,45]. Equimolar amounts of 5,5-dimethylhydantoin and KOH were dissolved in 100 mL ethanol. The mixture was heated at reflux until the solution became clear for about 10 min. After removal of the ethanol and water by evaporation of the solvents under reduced pressure, the potassium salt of 5,5-dimethylhydantoin was isolated. The collected salt was further dried at 60 °C for 4 days and obtained at 90% yield. Then, the dry potassium salt of 5,5-dimethylhydantoin was reacted with an equivalent amount of 3-chloropropyltriethoxysilane in 100 mL dimethyl formamide (DMF) at 95 °C for 4 h. The KCl produced in the reaction was removed by filtration, and the DMF solvent was removed by vacuum distillation. Finally BA-1 was obtained as a viscous oil liquid and identified as the desired precursor monomer. ^1^H-NMR (d-CDCl_3_): δ 0.41 (2H), 1.02 (9H), 1.23 (6H), 1.53 (2H), 2.71–2.79 (2H), 3.63 (6H), 7.29 (1H) (Figure S2 in Supplementary Materials).

#### 3.2.3. Synthesis of 1-Chloro-2,2,5,5-tetramethyl-4-imidazolidinone (MC)

The synthesis of MC compound has been described elsewhere [20]. Briefly, 2,2,5,5-tetramethyl-4-imidazolidinone (14.2 g, 0.1 mol) was dissolved in 100 mL of 1 N sodium hydroxide solution (0.1 mol). The mixture was stirred at 10 °C, and chlorine gas was bubbled into the solution until the pH reached 7. The precipitated white solid product was obtained by filtration and was recrystallized from a hexane/ether mixture. ^1^H-NMR (d-CDCl_3_): δ 1.34 (6H), 1.46 (6H), 7.85 (1H) (Figure S3 in Supplementary Materials).

#### 3.2.4. Synthesis of 2-Acrylamido-2-methyl-1-(5-methylhydantoinyl)propane (HA) and Hydantoin Acrylamide Siloxane Copolymer (HASL)

In order to synthesize HASL copolymer, first the precursor HA monomer was prepared according to a method outlined previously [33]. The Bucherer–Berg reaction was used for the synthesis of the HA monomer in which the hydantoin ring formed. A similar approach, forming a hydantoin ring from a ketone moiety, was previously used to synthesize N-halamine monomers [46] and polymers [31]. Briefly, 2-acrylamido-2-methyl-1-(5-methylhydantoinyl) propane (HA) was synthesized by reacting 2-acrylamido-2-methyl-4-pentanone, potassium cyanide, and ammonium carbonate in a 1:2:6 molar ratio in a water/ethanol (1:1 by volume) solvent mixture in a round flask at room temperature for 5 days. After evaporation of ethanol, the crude product was isolated by adding dilute HCl and collected as a white powder after filtration. HA precursor was recrystallized in acetonitrile and dried at 45 °C for 24 h prior to the HASL copolymer reaction. The molecular weight of HA was measured by mass spectrometry to be 239.128 g/mol, in accord with the calculated molecular weight for HA of 239.127 g/mol [32]. HA has a melting point of 178 °C.

The hydantoin acrylamide siloxane copolymer (HASL) was synthesized by free radical polymerization. 10 mmol of HA and of 10 mmol of 3-(trimethoxysilyl)propyl methacrylate (SL) were dissolved in 15 mL methanol, and 0.05 g AIBN (2,2-Azobis-2-methylpropionitrile) was added into the mixture. After nitrogen was bubbled into the mixture for 15 min to remove any dissolved oxygen, the mixture was heated to 65 °C, and reaction was continued for 2 h. After evaporation of the solvent, the copolymer was obtained as white solid. ^1^H-NMR (DMSO-*d*_6_): δ 0.6 (2H), 0.91 (3H), 1.25 (8H), 1.61 (1H), 1.88 (2H), 2.12 (2H), 2.17 (2H), 2.51 (2H), 3.48 (9H), 4.02–4.13 (2H), 7.59 (1H), 7.83 (1H), 10.59 (1H) (Figure S4 in Supplementary Materials). The HA mole fraction in the copolymer was calculated by comparing the signal area of total methyl group protons (Figure S4, 3.6 ppm) attached to the silicon atom in SL (-Si-(OCH_3_)_3_) to the imide proton signal area (Figure S4, 10.6 ppm) of the HA moiety. The reactivity ratio of m_HA_/_(_m_HA_ + m_SL)_ was calculated as 0.40. Thus the reactivity of SL was found to be slightly higher than that of HA when the feed ratio of monomers was 1:1. The reactivity of the monomers was observed to be similar to that reported previously [32]. In previous studies the intrinsic viscosity of the HASL copolymer (1:1 feed ratio) was reported to be 0.55 dL/g (in 2-methoxyethanol at 25 °C) [32].

### 3.3. Preparation of N-Halamine-Modified Wound Dressings

Synthesized BA-1 was dissolved in EtOH/H_2_O (1/1 *w*/*w*) at a concentration of 7.5 wt %. Wound dressings in the size of 10 cm × 10 cm were soaked in the coating solution for 15 min and then uniformly padded through a laboratory wringer (Birch Brothers Southern, Waxhaw, NC, USA). BA-1 coated dressings were then cured at 95 °C for 40 min and at 145 °C for 20 min. After curing they were soaked in 300 mL 0.25 (*w*/*v*) % AATCC detergent aqueous solution for 15 min, washed with copious amount of tap water and finally rinsed several times with DI water. Coated dressings were allowed to dry completely at 45 °C for 3–4 h before chlorination.

Wound dressings were coated with 5 wt % of Hy-Ep solutions prepared in acetone/H_2_O (1/1 *w*/*w*). As in the previous method, samples were uniformly padded through a laboratory wringer after soaking in Hy-Ep solutions for 15 min. Hy-Ep coated pads were then cured at 95 °C for 1 h and then at 145 °C for 20 min. Cured samples were washed and rinsed in detergent solution in the same manner as above in order to remove the unreacted compounds.

1-Chloro-2,2,5,5-tetramethyl-4-imidazolidi-none (MC) was used as an antimicrobial coating for the wound dressings. MC (at 1 wt %) was dissolved in ethanol solution at room temperature, and then the dressings were soaked in the coating solution for 15 min. The samples were padded through a laboratory wringer (Birch Brothers Southern, Waxhaw, NC, USA). MC-coated dressings were dried at room temperature for 24 h. Alternatively, they could also be dried at 45 °C for 2 h. In contrast to the other coating processes described here for the other N-halamine compounds, MC compound used for the coating solutions was chlorinated during the synthesis of the MC; therefore, after the coating process, no extra chlorination was necessary.

Wound dressings were coated in 3 wt % HASL copolymer solutions, which were prepared in EtOH/water (3/1 *w*/*w*). HASL copolymer-coated wound dressings were then cured at 145 °C for 1 h. After curing, the HASL-coated samples were washed and dried prior to chlorination as described above.

### 3.4. Chlorination Procedure

Precursor N-halamine-treated wound dressings were rendered antimicrobial by a chlorination process. Samples were chlorinated in dilute household bleach (10 *v*/*v* % of 8.25% commercial sodium hypochlorite solution) at pH 7 at room temperature for 30 min. Chlorinated samples were then washed thoroughly with distilled water and dried at 45 °C for 2 h to remove free chlorine from the surface of the wound dressings. The oxidative chlorine loadings on the dressings were determined by an iodometric/thiosulfate titration procedure. The weight percentage of the bound oxidative chlorine was calculated according to following formula

(1) $${\mathrm{Cl}}^{+}\text{\%}=\frac{35.45\times N\times V}{2\times W}\times 100,$$

where Cl^+^ % is the weight percent of oxidative chlorine on the samples, N and V are the normality (equiv/L) and volume (L) of the titrant (Na_2_S_2_O_3_), respectively, and W is the weight of the sample (g) used for the titration.

### 3.5. Shelf Life Stability Testing

The storage or shelf life stability of the oxidative chlorine bound onto the wound dressings by the chlorination procedure was evaluated. Wound dressings were stored in sealed opaque packaging in a cabinet (dark environment) at room temperature. The stability of the chlorine content over time was measured for up to 24 weeks. The stabilities of the N-halamine-coated dressings were determined by measuring the amount of remaining chlorine on the samples by using the standard iodometric/thiosulfate titration procedure as discussed above.

### 3.6. Antimicrobial Efficacy Testing

Two types of tests were conducted in order to determine the biocidal efficacies of the N-halamine-coated wound dressings. *Staphylococcus aureus* (*S. aureus*, ATCC 6538) was used as a Gram-positive bacterium and *Pseudomonas aeruginosa* (*P. aeruginosa*, ATCC 27853) was used as a Gram-negative bacterium in order to challenge the un-chlorinated and chlorinated-coated wound dressings and commercial silver alginate and PHMB dressings.

In the first method, a “sandwich test” was used [39]. In this procedure, both *S. aureus* and *P. aeruginosa* were suspended in 100 μM phosphate buffer (pH 7) to produce a suspension of known population (colony forming units, CFU). Then, an aliquot of 25 μL of this suspension was placed in the center of a swatch, at size of 2.54 cm × 2.54 cm, and a second identical swatch was placed on top. Both swatches were covered by a sterile weight to ensure a good contact with the bacteria. After predetermined contact times, samples were quenched by 5.0 mL of sterile 0.02 N sodium thiosulfate solutions to neutralize the oxidative chlorine and thus terminate the disinfection action. Samples were vortexed for 2 min, and then serial dilutions were prepared using pH 7, 100 μM phosphate buffer solutions which were plated on trypticase soy agar plates. After the plates were incubated at 37 °C for 24 h, viable bacterial colonies were counted for the biocidal efficacy analysis. All experiments were performed at least twice (on different days) using different bacterial inocula.

### 3.7. Zone of Inhibition Antibacterial Test

Zone of inhibition of the N-halamine-coated wound dressings were evaluated by the Kirby-Bauer testing technique. A modified version of the American Association of Textile Chemist and Colorist (AATCC 147) method was applied, and the samples were tested against *S. aureus* and *P. aeruginosa*. In this method *S. aureus* and *P. aeruginosa* were suspended in 100 μM phosphate buffer (pH 7) to produce a suspension of known population (approximately 10^7^ CFU). Then, an aliquot of 100 μL of this suspension was plated on trypticase soy agar plates. N-halamine-coated wound dressings were cut into 0.79 cm^2^ disks and placed onto the agar plates and gently pressed to ensure full contact. Control disks were prepared in the same manner and placed onto agar plates. The plates were incubated at 37 °C for 24 h. After the plates were incubated, the formed zones of inhibition (if any) around the disks were measured. Two replicates of the samples were examined.

### 3.8. Cytotoxicity of N-Halamine-Treated Wound Dressings

In vitro cytocompatibilities of N-halamine-treated dressings as well as commercial silver alginate and PHMB dressings were evaluated. The influence of the antimicrobial compounds on cell growth and viability was examined after 24 h incubation in the presence of antimicrobial N-halamine-treated and antimicrobial silver and PHMB commercial dressings by a direct contact test. For this test NIH 3T3 mouse fibroblast cells were cultured in DMEM media with 10% NCS and 1% Pen/Strep at 37 °C and 5% CO_2_. All samples were cut into disks (0.32 cm^2^) to fit into 96-well tissue culture plates. Prior to testing, the disk samples were sterilized by UV exposure for 15 min. Untreated wound dressing samples were used as negative controls. Unchlorinated precursor-treated and chlorinated N-halamine-treated materials were used as test samples. Individual specimens of the test samples were placed on the bottoms of the 96-well plates. Then, 10^4^ of NIH-3T3 mouse fibroblast cells were seeded onto each specimen. After 24 h of contact time for cell attachment, MTT assays were performed to quantify the cell viability using a micro-plate reader. Optical density values of the viable cell medium were recorded at 540 nm. Cell culture plates (TCPS) and untreated samples were used as controls. The cell viability was determined as the percentage compared to the untreated sample controls. The test was repeated twice with a total of 12 replicates recorded for each treatment. The results were presented as means ± standard errors.

### 3.9. Skin Irritation Testing

In vitro skin irritation of the MC-impregnated wound dressings was tested using the EpiDerm™ reconstructed human epidermal (RHE) model (ISO10993-10). A reconstructed human epidermal model EpiDerm™ kit (EPI-200-SIT) was purchased from MatTek Corporation (Ashland, MA, USA), and the Epiderm™ skin tissue inserts were transferred into 6-well plates and were conditioned overnight at 37 °C, 1% CO_2_, 95% RH (relative humidity), following the instructions of the manufacturer. Untreated and MC-impregnated dressings were cut into 0.32 cm^2^ disks, and two layers of each sample were placed on top of the EpiDerm™ skin tissue. Then, 25 μL of D-PBS (Dulbecco’s Phosphate Buffer Saline) was added onto the test specimens to moisten the skin tissue surface as described in the protocol for solid samples. 30 μL of D-PBS were used as a negative control, and 5% SLS (sodium lauryl sulfate) solution was used as a positive control. Three EpiDerm™ skin tissues were used for each test material (untreated and MC-treated samples), as well as for the positive control and a negative control. After 60 min exposure, the test samples were discarded, and the EpiDerm™ skin tissues were rinsed 15 times with D-PBS and washed with copious amount of saline solution. After 42 h of post-incubation, the EpiDerm™ skin tissue inserts were transferred into a 24-well plate prefilled with 300 µL of MTT [3-(4,5-Dimethylthiazole-2-yl)-2,5-diphenyl tetrazolium] (1 mg/mL) and incubated for 3 h. Then MTT medium was discarded, and the tissue inserts were extracted with 2 mL of isopropyl alcohol. Then 200 µL of the MTT extracts were transferred into 96-well plates, and the optical density (OD) of the extract was measured at a wavelength of 540 nm using a microplate reader. For each chemical-treated epidermis, the % viability was expressed as [(OD_sample_ − OD_blank_)/(OD_control_ − OD_blank_)] × 100. 

## 4. Conclusions

This study has revealed that N-halamine chemistry offers significant potential for producing an antimicrobial wound dressing. Hy-Ep and BA-1 monomer precursors, HASL polymer precursor and MC compound were successfully coated onto or impregnated into (in the case of MC) wound dressings, and these N-halamine-treated dressings were rendered antimicrobial by a simple chlorination process with a dilute sodium hypochlorite solution (MC was chlorinated during its synthesis). MC-treated wound dressings were stable to loss of oxidative chlorine when they were stored in darkness and also under florescent light for 6 months. Other N-halamine treated dressings showed less stability over time with BA-1-Cl being the second most stable treatment among the N-halamine compounds. Their shelf life stabilities would be improved upon storage in opaque packaging. Zone of inhibition tests performed to determine any leaching of the antimicrobials from the N-halamine-employed wound dressing materials demonstrated that there was no leaching of the oxidative chlorine from three of the samples (Hy-Ep-Cl, BA-1-Cl and MC) and only minimally from HASL-Cl. Antimicrobial efficacies of the materials were determined by a sandwich test in which viable bacteria were quantified. The N-halamine treated wound dressings exhibited a complete inactivation of the bacteria within 15 to 60 min against *S. aureus* (Gram-positive bacteria) and *P. aeruginosa* (Gram-negative bacteria). In addition, the N-halamine-treated dressings showed rapid inactivation rates when compared to commercially available silver alginate dressings. In vitro cytotoxicity tests designed to show potential toxicity of the N-halamine compounds indicated that they did not inhibit cell viability. In fact, the potential toxicity of the N-halamine compounds was observed to be insignificant and less toxic when compared to commercially available Ag and PHMB dressings. MC-treated wound dressings showed no skin irritation, as indicated by 100% skin cell viability. All factors considered, compound MC would seem to be the optimum N-halamine compound for employment in a wound dressing. It is inexpensive, available commercially, easily applied to wound dressings, stable when stored in opaque packaging, effective in the inactivation of pathogenic bacteria in brief contact times, and it shows minimal skin sensitivity in wound dressing materials.

## Figures and Tables

**Table 1:** Storage stability of MC, BA-1-Cl, HASL-Cl, and Hy-Ep-Cl under dark and fluorescent light conditions.

Time (Weeks)	* MC (Cl^+^ %)		* BA-1 (Cl^+^ %)	* HASL (Cl^+^ %)	* Hy-Ep (Cl^+^ %)
	Dark	Light	Dark	Dark	Dark
0	0.20	0.22	0.21	0.19	0.23
1	0.19	0.22	0.20	0.18	0.20
2	0.18	0.22	0.21	0.18	0.18
3	0.20	0.20	0.22	0.15	0.14
4	0.21	0.20	0.20	0.16	0.15
5	0.19	0.20	0.24	0.14	ND
6	0.20	0.20	0.18	0.13	ND
7	0.19	0.19	0.23	0.14	ND
8	0.19	0.19	0.20	0.14	0.17
9	0.20	ND	0.18	0.14	ND
10	0.19	0.18	0.19	0.16	0.14
11	ND	ND	0.18	0.14	0.13
12	ND	ND	0.17	ND	ND
13	ND	ND	0.17	0.13	ND
15	ND	ND	0.17	0.12	0.15
16	ND	0.13	0.18	0.11	0.15
17	0.18	0.13	0.19	ND	0.13
18	0.21	0.13	ND	ND	ND
19	0.18	0.14	ND	ND	ND
20	0.17	0.16	ND	ND	ND
21	0.19	0.16	ND	ND	ND
22	0.21	0.13	ND	ND	ND
23	0.19	0.15	ND	ND	ND
24	0.21	0.15	0.16	0.07	0.06

* The average deviation in the measured Cl^+^ loading was ±0.03%. Each datum represents an average of three samples. Chlorine loadings are reported in wt % Cl^+^. ND represents no determination.

**Table 2:** Biocidal efficacy of MC-treated wound dressings.

Samples	Contact Time (min)	Log Reduction * Exp 1		Log Reduction * Exp 2		Log Reduction * Exp 3	
Inoculum		6.07	7.36	6.05	6.11	6.36	5.70
		*S. aureus*	*P. aeruginosa*	*S. aureus*	*P. aeruginosa*	*S. aureus*	*P. aeruginosa*
Untreated dressing (control)	60	0.18	2.13	0.04	1.26	0.20	0.98
MC-treated dressing Cl^+^ = 0.23 wt %	5	6.07	2.06	6.05	0.28	2.03	0.89
	15	6.07	5.24	6.05	2.30	6.36	2.97
	30	6.07	7.36	6.05	6.11	6.36	5.70
	60	6.07	7.36	6.05	6.11	6.36	5.70

* Data represent three different experiments each being performed on different days with different inoculum concentrations. Average Cl^+^ % for all three experiments was measured as 0.23.

**Table 3:** Biocidal efficacy of BA-1-Cl coated wound dressings.

Samples	Contact Time (min)	Log Reduction * Exp 1		Log Reduction * Exp 2		Log Reduction * Exp 3	
Inoculum		6.07	7.36	6.05	6.11	6.36	5.70
		*S. aureus*	*P. aeruginosa*	*S. aureus*	*P. aeruginosa*	*S. aureus*	*P. aeruginosa*
Untreated dressing (control)	60	0.05	0.62	0.31	1.26	0.006	0.91
BA-1-Cl-treated dressing Cl^+^ = 0.21 wt %	5	0.56	0.98	3.45	0.71	0.032	0.029
	15	3.64	1.46	2.78	6.11	2.53	0.494
	30	3.94	1.79	3.45	6.11	6.36	2.57
	60	3.54	4.94	3.08	6.11	6.36	5.70

* Data represent three different experiments each being performed on different days with different inoculum concentrations. Average Cl^+^ % for all three experiments was measured as 0.21.

**Table 4:** Biocidal efficacy of Hy-Ep-Cl coated wound dressings.

Samples	Contact Time (min)	Log Reduction * Exp 1		Log Reduction * Exp 2		Log Reduction * Exp 3	
Inoculum		6.07	7.36	6.05	6.11	6.00	6.22
		*S. aureus*	*P. aeruginosa*	*S. aureus*	*P. aeruginosa*	*S. aureus*	*P. aeruginosa*
Untreated dressing (control)	60	0.19	2.33	0.65	3.69	0.17	0.68
Hy-Ep-Cl-treated dressing Cl^+^ = 0.20 wt %	5	0.33	0.91	0.10	0.13	0.79	0.02
	15	1.25	1.16	2.81	0.23	0.70	0.06
	30	2.45	1.46	2.14	2.39	6.00	0.55
	60	6.07	4.06	3.08	6.11	6.00	6.22

* Data represent three different experiments each being performed on different days with different inoculum concentrations. Average Cl^+^ % for all three experiments was measured as 0.20.

**Table 5:** Biocidal efficacy of HASL-Cl coated wound dressings.

Samples	Contact Time (min)	Log Reduction * Exp 1		Log Reduction * Exp 2		Log Reduction * Exp 3	
Inoculum		6.05	6.11	6.00	6.22	6.36	5.70
		*S. aureus*	*P. aeruginosa*	*S. aureus*	*P. aeruginosa*	*S. aureus*	*P. aeruginosa*
Untreated dressing (control)	60	0.02	1.42	0.04	1.26	0.20	0.98
HASL-Cl-treated dressing Cl^+^ = 0.23 wt %	5	2.97	3.99	1.34	0.76	2.03	0.89
	15	6.05	6.11	3.27	2.89	6.36	2.97
	30	6.05	6.11	6.00	6.22	6.36	5.70
	60	6.05	6.11	6.00	6.22	6.36	5.70

* Data represents three different experiments each being performed on different days with different inoculum concentrations. Average Cl^+^ % for all three experiments was measured as 0.23.

**Table 6:** Biocidal efficacy of commercial silver alginate and polybiguanide (PHMB) wound dressings.

Samples	Contact Time (min)	Log Reduction * Exp 1		Log Reduction * Exp 2	
Inoculum		7.11	7.09	7.39	7.07
		*S. aureus*	*P. aeruginosa*	*S. aureus*	*P. aeruginosa*
Untreated dressing (control)	30	0.23	0.62	0.08	0.17
Silver alginate-treated dressing	1	0.22	0.13	0.11	0.12
	5	0.14	0.18	0.15	0.31
	15	0.29	0.23	0.50	0.42
	30	0.36	0.31	0.98	1.14
PHMB-treated dressing	1	1.59	1.62	4.66	0.41
	5	3.32	2.05	4.78	1.31
	15	3.31	2.22	7.39	2.97
	30	3.63	3.45	7.39	2.58

* Data represents two different experiments being performed on different days with different inoculum populations.

## Supplementary Materials

Supplementary materials are available online. That contains spectral data confirming the structures of the synthesized materials and zone of inhibition data for the wound dressings.
