# Supplementary material for: N-Halamine Biocidal Materials with Superior Antimicrobial Efficacies for Wound Dressings
Source: Molecules. 2017 Sep 21;22(10):1582. doi: 10.3390/molecules22101582 (PMC6151735; doi:10.3390/molecules22101582)
Supplement: Supplementary file 1 [file molecules-22-01582-s001.pdf]

Supplementary Materials

N-Halamine Biocidal Materials with Superior Antimicrobial Efficacies for Wound Dressings

Buket Demir [1], Roy M. Broughton [2], Mingyu Qiao [3], Tung-Shi. Huang [3], S. D. Worley [1,*]

[1] Department of Chemistry and Biochemistry, Auburn University, Auburn, AL 36849; bzd0003@tigermail.auburn.edu (B. D.); worlesd@auburn.edu (S.D.W.)

[2] Center for Polymers and Advanced Composites, Department of Mechanical Engineering, Auburn University, Auburn, AL, 36849; brougrm@auburn.edu (R.M.B.)

[3] Department of Poultry Science, Auburn University, Auburn, AL, 36849; huangtu@auburn.edu (T-S. H.); mzq0001@tigermail.auburn.edu (M. Q.)

* Correspondence: worlesd@auburn.edu (S.D.W); Tel.: +01-334-844-6980 (S.D.W.)

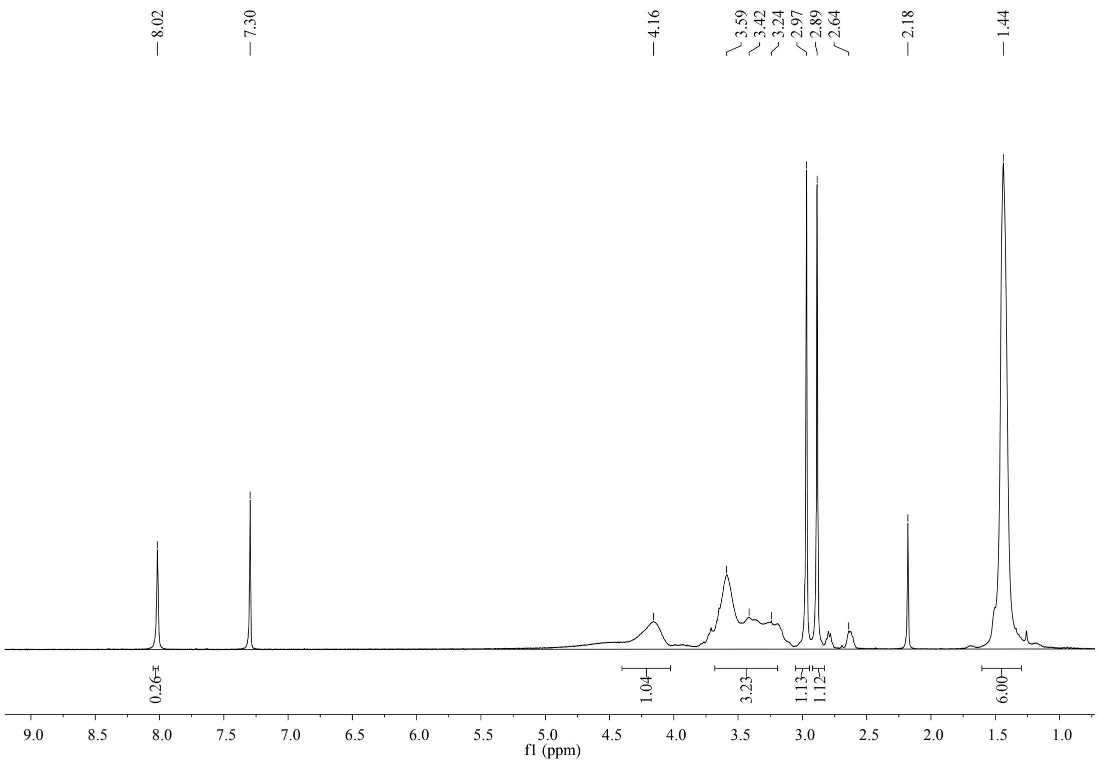

Figure S1. ^{1}H-NMR (d-$CDCl_3$) of the 3-Glycidyl-5,5-dimethylhydantoin (Hy-Ep).

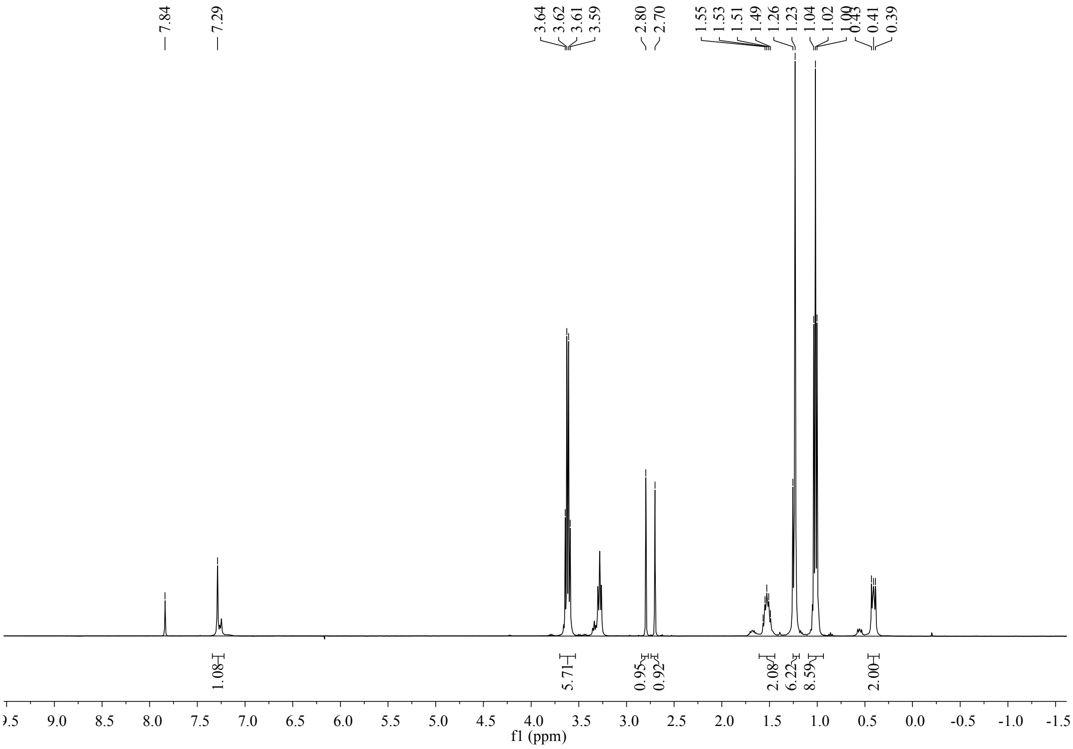

Figure S2. ^{1}H-NMR (d-$CDCl_3$) of the 3-triethoxysilylpropyl-5,5-dimethylhydantoin (BA-1).

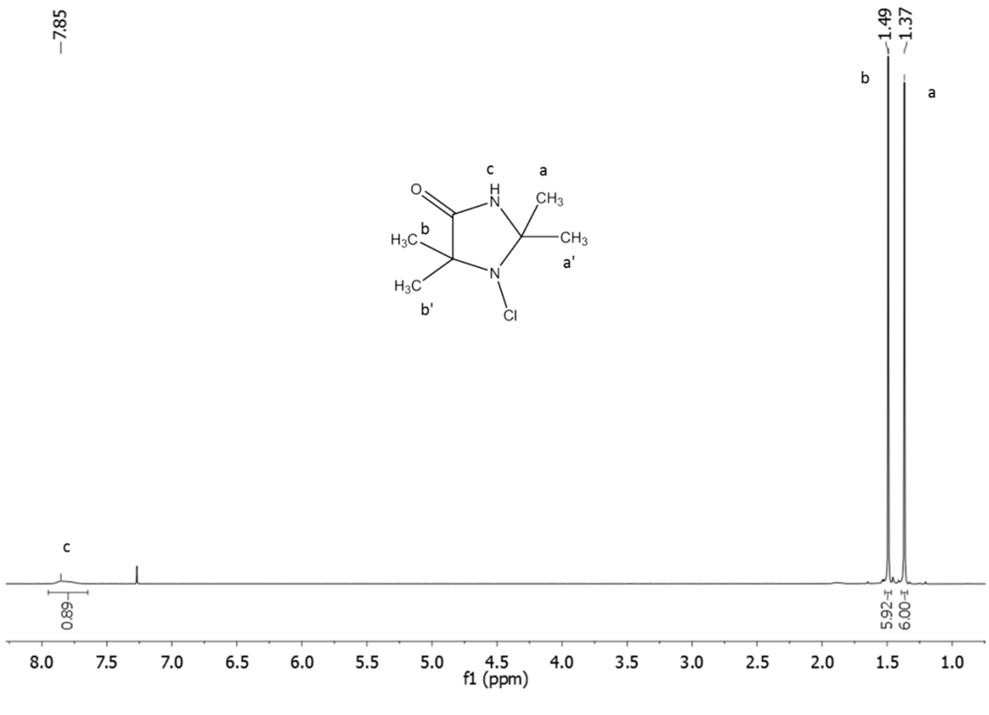

Figure S3. ^{1}H-NMR (d-$CDCl_3$) of the 1-chloro-2,2,5,5-tetramethyl-4-imidazolidinone (MC).

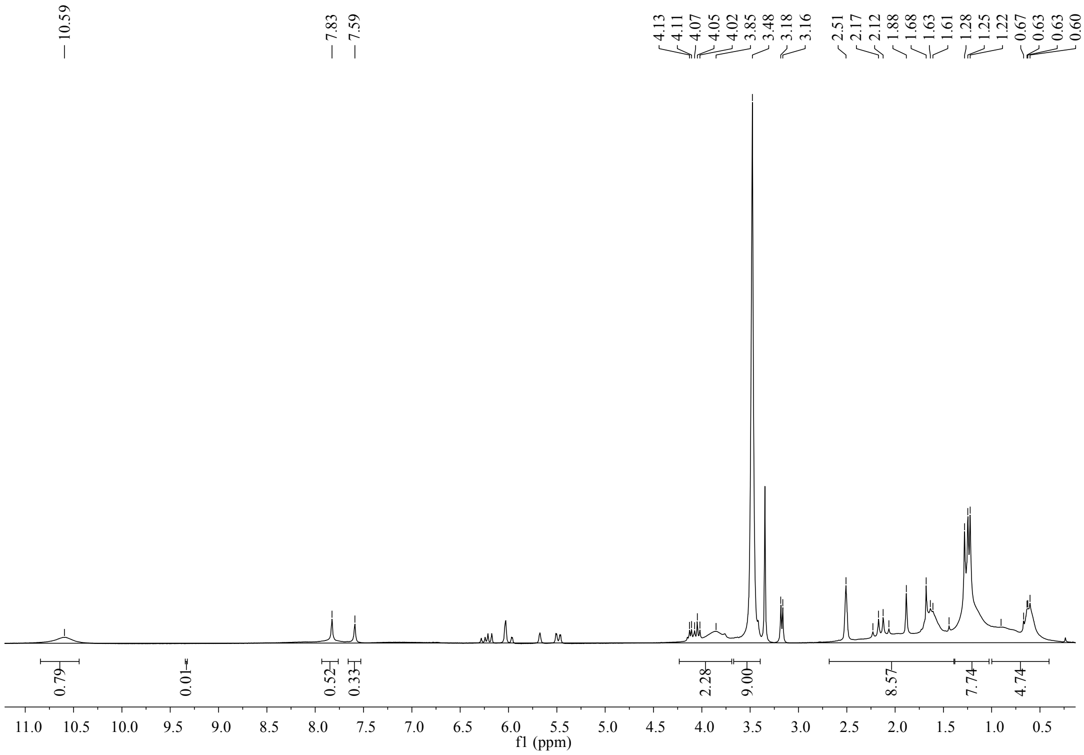

Figure S4. ^{1}H-NMR (DMSO-d_6) of the hydantoin acrylamide siloxane copolymer (HASL).

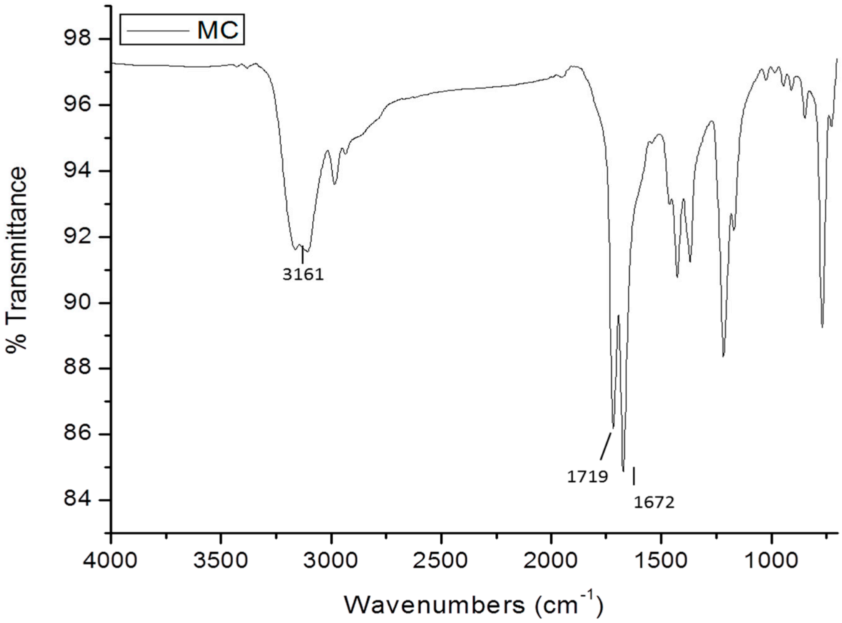

Figure S5. FTIR spectra of the 1-chloro-2,2,5,5-tetramethyl-4-imidazolidinone (MC).

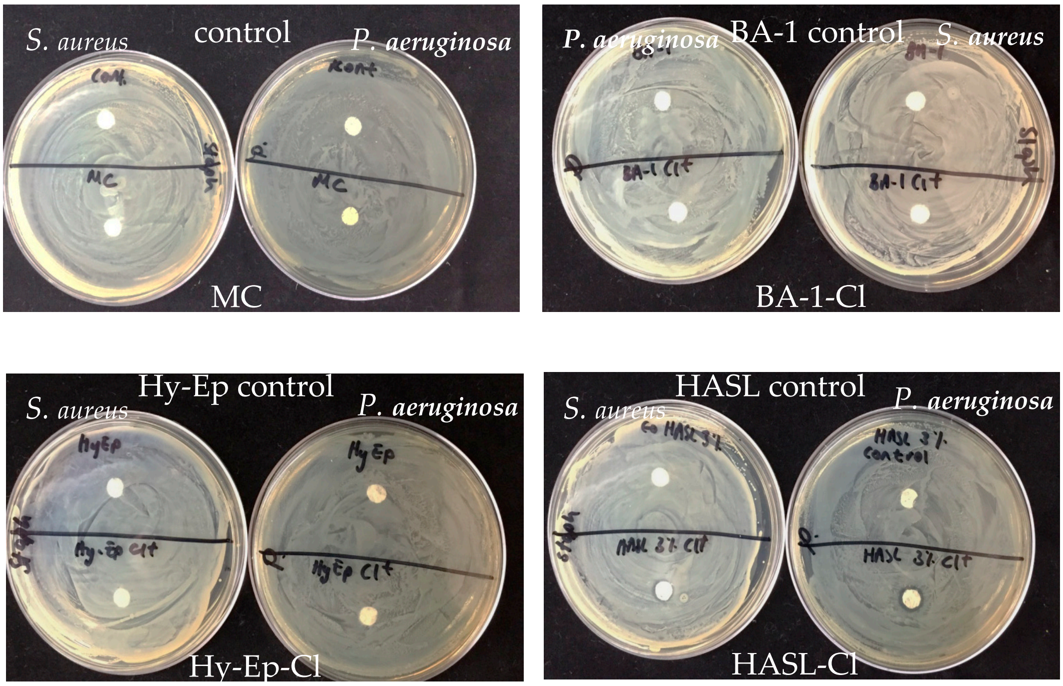

Figure S6. Zone of inhibition of a) MC b) BA-1-Cl c) Hy-Ep-Cl and d) HASL-Cl treated dressings, unchlorinated disks were shown on the top half part of each agar plate and N-halamine treated and chlorinated disks were shown on the bottom half of each agar plate against *S. aureus* (left plate) *and P. aeruginosa* (right plate).
